# Estimating Premorbid Cognitive Abilities in Low-Educated Populations

**DOI:** 10.1371/journal.pone.0060084

**Published:** 2013-03-21

**Authors:** Daniel Apolinario, Sonia Maria Dozzi Brucki, Renata Eloah de Lucena Ferretti, José Marcelo Farfel, Regina Miksian Magaldi, Alexandre Leopold Busse, Wilson Jacob-Filho

**Affiliations:** 1 Geriatrics Division, Department of Internal Medicine, University of São Paulo Medical School, São Paulo, São Paulo, Brazil; 2 Behavioral and Cognitive Neurology Unit, Department of Neurology, University of São Paulo Medical School, São Paulo, São Paulo, Brazil; 3 Medical-Surgical Nursing Department, University of São Paulo School of Nursing, São Paulo, Brazil; 4 Brazilian Brain Bank of the Aging Brain Study Group, University of São Paulo Medical School, São Paulo, Brazil; Nathan Kline Institute and New York University School of Medicine, United States of America

## Abstract

**Objective:**

To develop an informant-based instrument that would provide a valid estimate of premorbid cognitive abilities in low-educated populations.

**Methods:**

A questionnaire was drafted by focusing on the premorbid period with a 10-year time frame. The initial pool of items was submitted to classical test theory and a factorial analysis. The resulting instrument, named the Premorbid Cognitive Abilities Scale (PCAS), is composed of questions addressing educational attainment, major lifetime occupation, reading abilities, reading habits, writing abilities, calculation abilities, use of widely available technology, and the ability to search for specific information. The validation sample was composed of 132 older Brazilian adults from the following three demographically matched groups: normal cognitive aging (n = 72), mild cognitive impairment (n = 33), and mild dementia (n = 27). The scores of a reading test and a neuropsychological battery were adopted as construct criteria. Post-mortem inter-informant reliability was tested in a sub-study with two relatives from each deceased individual.

**Results:**

All items presented good discriminative power, with corrected item-total correlation varying from 0.35 to 0.74. The summed score of the instrument presented high correlation coefficients with global cognitive function (r = 0.73) and reading skills (r = 0.82). Cronbach's alpha was 0.90, showing optimal internal consistency without redundancy. The scores did not decrease across the progressive levels of cognitive impairment, suggesting that the goal of evaluating the premorbid state was achieved. The intraclass correlation coefficient was 0.96, indicating excellent inter-informant reliability.

**Conclusion:**

The instrument developed in this study has shown good properties and can be used as a valid estimate of premorbid cognitive abilities in low-educated populations. The applicability of the PCAS, both as an estimate of premorbid intelligence and cognitive reserve, is discussed.

## Introduction

An individual's previous cognitive performance is the standard against which current performance should be compared to identify cognitive decline. Because most patients do not have records of previous functioning, estimates of premorbid intelligence constitute an essential aspect of neuropsychological assessment [1]. Such estimates have also provided grounds for research on cognitive reserve [2]. The cognitive reserve hypothesis proposes that lifetime intellectual enrichment attenuates the negative impact of neurologic disease on cognitive status [3].

One approach to estimate premorbid intelligence and cognitive reserve involves the use of demographic characteristics such as educational attainment and lifetime occupation [4]. These methods can be useful because the data are easy to acquire and remain constant without being affected by any cognitive decline that may have occurred. However, demographic characteristics provide limited estimates of cognitive functioning, reflecting the fact that intellectual development occurs beyond those factors and continues throughout life [5]–[7].

Some methods have been proposed to estimate premorbid intellectual functioning by assessing over-learned abilities that are more resistant to the effects of cerebral dysfunction. The most common approach is the use of reading tests, which require the participant to read aloud words with irregular pronunciation or to identify real words from pseudo-words [8]–[11]. In contrast to demographic approaches, reading tests are more highly correlated with cognitive performance, but some studies have found that reading skills are not entirely impervious to the effects of cognitive impairment [12]–[15].

A more recent approach is the use of questionnaires developed to rate an individual's level of participation in cognitively demanding activities. In the United States, Wilson et al. validated a self-report questionnaire to rate the frequency of participation in six activities at five life stages, exploring mainly reading and writing habits [16]. Schinka et al. derived and validated a 25-item scale to rate an individual's level of engagement in a wide range of cognitively stimulating activities, such as playing games, taking courses, attending social events, and practicing artistic activities [17]. In Australia, Valenzuela et al. validated an instrument examining information from three life stages, which included questions about leisure activities, fitness pursuits, educational attainment, and occupation [18]. Recently, three questionnaires were validated in European countries, with rationales and structures that are similar to the preceding instruments [19]–[21].

Cognitive reserve questionnaires show moderate to strong correlations with measures of cognitive performance and reading skills [17], [19], [21] but have encountered some limitations from a practical perspective. Considerable changes in activity participation occur within short periods of time and can be affected by a variety of transient factors such as illness or job relocation. Thus, the strategy of rating the frequency of participation in specific activities across different stages of life may induce recall errors and reduce reliability [22]. By requiring detailed information about different periods of someone's life, these questionnaires are suitable only for the context of self-report, making their use unrealistic in patients with dementia or in post mortem studies. Additionally, questionnaire-based instruments have been designed and validated only in developed countries, with samples invariably averaging above nine years of schooling. As long as they assess activities that are highly dependent on the socioeconomic environment, these instruments have important drawbacks in low-educated populations who have grown up with limited opportunities and are seldom engaged in leisure activities [23].

In populations with low educational levels, we hypothesize that questions about basic skills like reading, writing, calculation, and the use of simple technology would constitute a more suitable assessment of premorbid cognitive functioning. We also propose that, in contrast to rating the frequency of participation, asking about easily observable abilities with yes/no answers would attenuate recall errors and allow the use of collateral information. The purpose of the present study was to develop a practical informant-based scale that can be used to provide a valid estimate of premorbid cognitive abilities for low-educated populations.

## Methods

### Subjects

One hundred thirty-two participants were recruited from a geriatric memory clinic at the University of São Paulo Medical School, Brazil. Patients referred from July 2009 to February 2011 were screened for participation. To be eligible, the subject had to meet the following criteria: (1) age ≥60 years; (2) availability of a knowledgeable relative or close friend who had regular contact with the individual 10 years ago (saw him/her at least every month); and (3) absence of a hearing, vision, motor or speech impairments that precluded adequate interaction with the interviewer.

**Figure 1:**
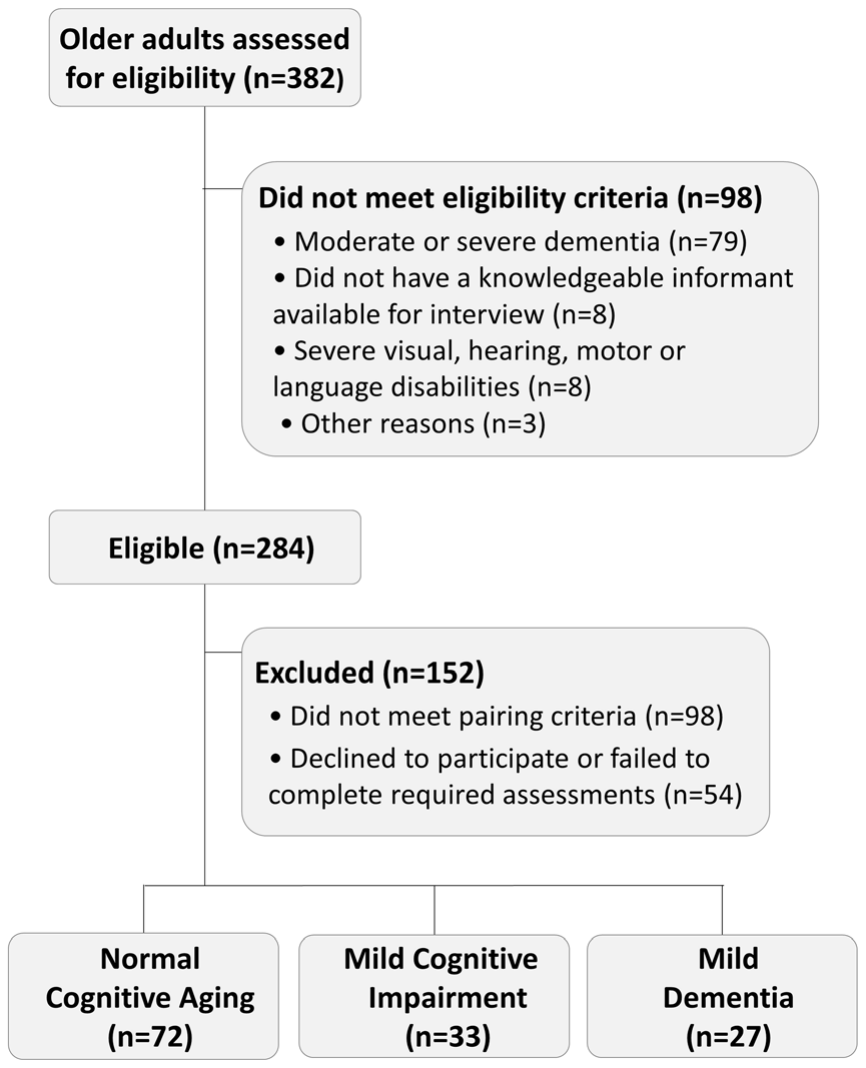
Flowchart depicting the steps involved in the recruitment process.

The control group was composed of 72 individuals with normal cognitive aging, as indicated by neuropsychological tests within the normal range and a Clinical Dementia Rating (CDR)  = 0. Two additional groups were composed of individuals with mild cognitive impairment (MCI; n = 33) and mild dementia (n = 27). Subjects with MCI had a CDR  = 0.5 and met the criteria described by the International Working Group on Mild Cognitive Impairment [24]. Subjects with mild dementia had a CDR  = 0.5 or 1 and fulfilled the Diagnostic and Statistical Manual of Mental Disorders, fourth edition (DSM-IV) criteria for dementia [25]. The MCI and mild dementia groups were closely matched to the control group on the basis of age, gender and education. Figure 1 depicts the steps involved in the recruitment process.

Participants with normal cognitive aging and MCI gave written consent before the interview. Because some subjects with mild dementia may have questionable capacity to consent, next of kin consented on behalf of the demented participants. The research protocol was approved by the Research Ethics Com­mittee of the *Hospital das Clínicas da Faculdade de Medicina da Universidade de São Paulo*.

To assess inter-informant reliability, a sub-study was conducted with 20 pairs of informants recruited at the São Paulo Autopsy Service between October 2011 and June 2012 according to the procedures of the Brazilian Brain Bank of the Aging Brain Study Group [26], [27]. Two relatives of each individual who had died within the preceding hours and who were waiting for the release of the body were invited to answer the final version of the questionnaire. The informants were required to provide answers independently without communicating with each other.

### Development of the questionnaire

Because self-report relies heavily on memory and is unlikely to be accurate in the presence of dementia, we developed a questionnaire to be completed by a close friend or relative. We focused on the premorbid period to ensure that our estimate was independent of possible cognitive decline. The 10-year time frame was chosen because the duration of dementia is usually less than 10 years. Longer time frames would make it difficult to find informants capable of providing accurate information. This strategy is common in instruments designed to rate individuals with known or suspected cognitive impairment [28], [29].

To maximize clinical utility, the instrument was designed with a self-explanatory structure for independent completion. Easily observable abilities were investigated with a yes/no answer system to avoid the burden of subjective judgment. We have favored activities with minimal physical and economic requirements to enhance the applicability among persons with diverse backgrounds. Candidate items were either formulated *de novo* or adapted from existing instruments [16]–[21]. In a pilot study, the 22 items initially drafted were given to family members of 20 elderly patients for comment on ease of understanding. The item pool was then refined by substitution or rewording in an iterative manner.

Because we had no previous knowledge of the relationship between reading habits and cognitive performance in this population, the two items planned to investigate reading habits were initially formulated as open ended questions, registered verbatim and transformed into standardized scores that were developed later. The questionnaire was composed of an additional 20 questions that accepted only dichotomous answers (yes/no). The questionnaire was given to the informants for self-completion along with the following instructions: “*Try to remember what your relative or friend was like 10 years ago. Check yes only if he or she was able to perform the task in question without any help.*”

### Demographic information

In low-educated populations, the effects of education on cognitive performance are not linear; rather, the relationship is a negatively accelerated curve that tends to reach a plateau [30]. Accordingly, the participants were grouped to impose a linear effect on the variable. By following this strategy, education was stratified into the following six levels: zero years; 1 year; 2–3 years; 4–7 years; 8–11 years; ≥12 years.

With the intention to obtain an ordinal variable deemed to represent rank-ordered levels, occupation was classified in one of the following categories: (1) rural unskilled manual workers; (2) urban unskilled manual workers (menial and repetitive tasks); (3) skilled manual workers (specific tasks that require training); (4) routine non-manual employees and self-employed workers; and (5) intellectuals, administrators and higher-degree technicians.

Economic status was determined according to the Brazilian Economic Classification Criterion [31], which provides a continuous scale calculated by assigning scores to the number of household assets. Participants were further classified by gender (male/female) and color assigned by the interviewer (white/non-white).

### Measures for construct validation

We adopted two criteria for assessing construct validity. Cognitive performance was chosen because its estimation is the main reason for the existence of premorbid intelligence measures. Reading ability was also chosen because it is a well-accepted method to estimate intelligence and cognitive reserve.

All participants were given a standardized neuropsychological battery known as NEUROPSI [32], [33], which is composed of 26 subtests developed to assess a wide spectrum of cognitive functions. By design, NEUROPSI includes items that are culturally appropriate for Latin American populations and can be applied to low-educated individuals. NEUROPSI is divided into the following sections: (1) Orientation (day, month, year, city, specific place, age); (2) Attention (digits backward, cancelation of figures, serial subtractions); (3) Visuospatial Skills (copy of a semi-complex figure); (4) Memory (a 6-word list presented 3 times, spontaneous recall of the word list, cued recall of the word list, recognition of the word list, recall of the semi-complex figure); (5) Language (naming line drawings, repetition of words and sentences, comprehension of commands, semantic verbal fluency, phonological verbal fluency); (6) Reading and Writing (answering questions after reading a paragraph, writing sentences by copying and dictation); (7) Conceptual Functions (similarities, everyday mathematical problems, completing sequences by deducing patterns); and (8) Motor Functions (changing the position of the hands, alternating movements, opposite reactions). The scores of these subscales can be summed to obtain a total score with a maximum of 130 points.

**Figure 2:**
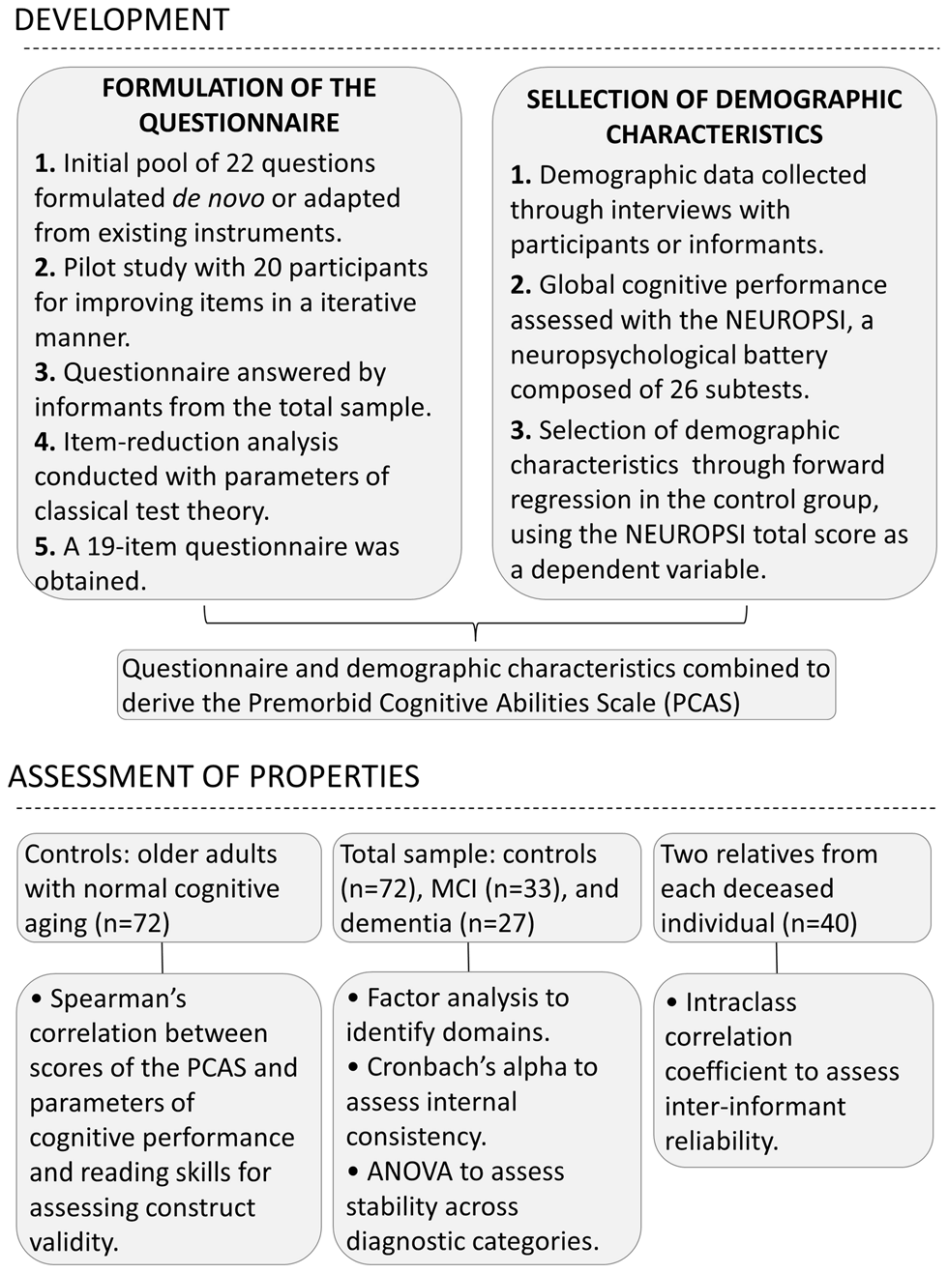
Flowchart depicting the steps involved in the development and validation of the PCAS.

Reading skills, including pronunciation and vocabulary, were tested with the Short Assessment of Health Literacy for Portuguese-Speaking Adults (SAHLPA) [34]. The SAHLPA requires the examinees to read aloud 50 common medical terms and choose, between two alternatives, the word that is most similar in meaning to the medical term. All interviews and assessments were conducted by the first author. Figure 2 depicts a flowchart with the steps involved in the development and validation of the scale.

### Statistical analysis

Analysis based on classical test theory was conducted to verify whether any poorly performing items of the questionnaire had to be dropped [35]. The procedures involved removing items with the following attributes: (1) proportion of positive answers ≤5% or ≥95% indicating floor or ceiling effects; (2) inter-item correlation ≥0.80 indicating redundancy; (3) corrected item-total correlation coefficient ≤0.30 indicating low discriminative power.

To investigate factor structure, a principal component analysis (PCA) was conducted by applying direct Oblimin rotation. An oblique rotation method was used because emerging factors were expected to be correlated. Eigenvalues were obtained for each component, a scree plot was derived for visual inspection, and Horn's parallel analysis was conducted to compare the size of the eigenvalues obtained from PCA with those obtained from a randomly generated dataset [36]. The PCA was repeated by fixing the number of factors encountered in parallel analysis, and the rotated pattern matrix was inspected by searching for items with loading values of 0.4 or greater.

To decide which socio-demographic variables should be included in the scale, we conducted a forward multiple regression analysis in the control group using the NEUROPSI total score as a dependent variable and the demographic data as independent variables.

Demographic variables were compared between diagnostic groups to appraise the effectiveness of the pairing procedure. To verify whether the scale is impervious to the effects of cognitive impairment, we examined whether the mean scores of the scale remain relatively stable across the diagnostic categories. Between-group comparisons were carried out using one-way analyses of variance (ANOVA) for parametric data or a Kruskal-Wallis test for non-parametric data, both followed by post-hoc multiple comparisons tests – Tukey's test and Conover's method, respectively.

Spearman's rank test was used to calculate the correlation coefficients because a normal distribution could not be demonstrated for all parameters studied. Internal consistency was examined using the Cronbach's alpha coefficient for each subscale and for all items together. Values over 0.7 were considered to be acceptable, and values over 0.9 were taken as indicative of redundancy. To access inter-informant reliability, we calculated the intraclass correlation coefficient (ICC) and its 95% confidence interval.

A power analysis based on the ANOVA indicated that our sample would provide 72% power for detecting between-group differences with medium effect sizes (*f* = 0.25). Analyses were conducted using the Statistical Package for Social Sciences version 17.0 (SPSS Inc., Chicago, IL) and power calculations were conducted with the software G*Power 3.1.5 [37]. All statistical tests were two-tailed, and an alpha level of less than 0.05 was used to indicate statistical significance.

## Results

### Development of the questionnaire

Of the initial pool, three items had to be eliminated. The items “*use money for payments*” and “*tell what time it is*” were eliminated for presenting a strong ceiling effect. The item “*read simple words*” was removed because of redundancy, indicated by a high correlation coefficient (r = 0.83) with the item “*read simple sentences*”. Corrected item-to-total correlations ranged from 0.35 to 0.74, and therefore, no item had to be excluded due to low discriminative power. The results of the stepwise item-reduction analysis are presented in Table 1.

**Table 1:** Classical test theory and principal component analysis results.

	Able to Perform the Task (%)	Item-Total Correlation	Factor Loadings on Subscale 1	Factor Loadings on Subscale 2
**SUBSCALE 1**				
Solve simple problems involving multiplication (times table)	73	0.73	0.47	0.47
Find information about a drug on package inserts	57	0.66	0.63	-
Read and understand an entire book	52	0.66	0.61	-
Use a calculator to solve simple problems	50	0.61	0.62	-
Solve problems involving percentage (price discount)	46	0.56	0.60	-
Used to read magazines or newspapers at least once per week	45	0.53	0.60	-
Find information about a device in the instruction booklet	39	0.58	0.74	-
Find a new location on a map	39	0.57	0.72	-
Use an ATM machine to withdraw cash	39	0.50	0.57	-
Used to read at least one book per year	39	0.41	0.60	-
Use a computer to type and print text	12	0.36	0.58	-
**SUBSCALE 2**				
Find a number in the phone book and make a call	93	0.40	-	0.66
Read and understand short sentences	92	0.54	-	0.89
Take note of a message	89	0.52	-	0.79
Write down a shopping list	87	0.51	-	0.81
Read and understand magazine reports	83	0.64	-	0.61
Fill out a form with personal data	80	0.67	-	0.59
Read and understand a medical prescription	76	0.62	-	0.44
Write a letter for someone else	71	0.68	0.42	0.45

The Kaiser-Meyer-Olkin (KMO) measure supported sampling adequacy for a PCA analysis (KMO = 0.90), and Bartlett's test of sphericity indicated that correlations between items were sufficiently large for PCA (p<0.001). Three components had eigenvalues over Kaiser's criterion of 1, but the scree plot showed an ambiguous inflection. Horn's parallel analysis identified a two-factor solution, which accounted for 50.0% of the total variance. Accordingly, the PCA was repeated by fixing the extraction of two factors. The clustering observed on the first factor was identified as “advanced cognitive abilities” and included 11 items. The second factor was identified as “basic reading and writing abilities” and clustered 8 items. Two items – “write a letter” and “solve problems involving multiplication” – were cross-loaded (Table 1).

On the forward multiple regression with socio-demographic variables, only education (p<0.001) and occupation (p = 0.02) were found to independently predict the NEUROPSI total score. The hierarchical multiple regression revealed that the questionnaire had a coefficient of determination (R^2^)  = 0.50. When education and occupation were simultaneously entered, there was a significant change in predictive power (F change  = 7.66; p = 0.007), with a final R^2^ = 0.55.

In accordance with the results described above, a final instrument was obtained with items addressing educational attainment (5 points), major lifetime occupation (4 points), reading abilities (4 points), writing abilities (4 points), use of technology (3 points), abilities to search for specific information (4 points), reading habits (4 points), and calculation abilities (2 points). The resulting instrument, named the Premorbid Cognitive Abilities Scale (PCAS), provides scores varying from 0 to 30. See Appendix S1 for the original version in Brazilian Portuguese. Appendix S2 presents a translation of the instrument into English, which has been accomplished by the authors and reviewed by a language editing service. The English version has been back-translated by a second language service and presented good semantic equivalence.

### Characteristics of the sample and of the informants

Our sample consisted of 132 older adults with a mean age of 73.6 (±7.2) years, 78% female, who had an average of 5.2 (±4.3) years of schooling. Although we have matched the sample for only three variables, the three groups did not differ significantly on socio-demographic and clinical variables, with the exception that economic status was slightly lower in individuals with MCI when compared with the other two groups (Table 2).

**Table 2:** Demographic and clinical characteristics of the study participants.

	Controls (n = 72)	MCI (n = 33)	Dementia (n = 27)	P Value	Post-hoc test
**Age** (years)	73.0 (7.8)	73.9 (5.7)	74.7 (7.2)	0.589*	
**Gender** (% female)	58 (80.6)	25 (75.8)	20 (74.1)	0.736#	
**Education** (years)	5.2 (4.3)	5.3 (4.2)	5.0 (4.4)	0.687†	
**Color** (% white)	53 (73.6)	22 (66.7)	19 (70.4)	0.762#	
**Marital Status** (% married)	38 (52.8)	15 (45.5)	12 (44.4)	0.671#	
**Economic Status** (CCEB)	22.3 (6.7)	18.9 (4.9)	23.8 (6.6)	0.014†	C>M<D
**Occupation** (% manual worker)	37 (51.4)	14 (42.4)	14 (51.8)	0.664#	
**Comorbidity** (CCI)	1.1 (1.0)	1.3 (1.3)	0.9 (1.0)	0.507†	
**Number of Drugs**	5.9 (3.3)	6.1 (3.2)	5.2 (3.1)	0.533*	
**Depressive Symptoms** (GDS-15)	5.0 (3.7)	5.5 (3.3)	3.8 (2.8)	0.141†	
**MMSE Score**	25.6 (3.4)	23.7 (4.7)	19.2 (3.7)	<0.001†	C>M>D
**CDR_SOB_**	0 (0)	1.7 (0.6)	5.1(1.7)	<0.001†	C<M<D

Data are shown as the mean (standard deviation) or number (percentage). Abbreviations: MCI, Mild Cognitive Impairment; CCI, Charlson Comorbidity Index; GDS-15, Geriatric Depression Scale – 15 items; CDR_SOB_, Clinical Dementia Rating Sum of Boxes; MMSE, Mini-Mental State Examination; * ANOVA; # Chi-square test; † Kruskal-Wallis test. “C>M” means that C is significantly higher than M.

The informants had a mean age of 51.4 (±15.3) years, 76.7% were female, and they had an average of 9.8 (±4.5) years of schooling. They were mostly children (54.5%), spouses (21.2%), friends (9.1%), and siblings (5.3%) of the patient. Over half (58.3%) lived with the patient. In eight (6.1%) cases, the questionnaire had to be completed by a telephone interview. Of the 124 informants who attended the interview meeting, only six (4.8%) needed significant help to complete the questionnaire.

Informants for the reliability sub-study consisted of 23 children, eight grandchildren, five siblings, three spouses, and one niece. Of the 20 deceased subjects who had their abilities rated, 18 presented normal cognitive aging (CDR = 0), one had questionable dementia (CDR = 0.5) and one had mild dementia (CDR = 1). Their mean age was 72.0 (±13.6) years, 50.0% were female, and they had an average of 3.1 (±2.8) years of schooling.

### Performance of the questionnaire

The score of the PCAS ranged from 0 to 30, with a mean of 16.4 (±7.1). The non-significant Kolmogorov-Smirnov test (p = 0.307) suggests normality of the distribution. The coefficient of skewness indicated a slight clustering of scores at the high end (−0.420; p = 0.048).

The correlation of the scale being validated with the adopted construct criteria was good to excellent. Spearman's coefficient between the PCAS and the NEUROPSI total score was 0.73 in the control group, varying according to domain from 0.36 (orientation) to 0.74 (language). Spearman's coefficient between the PCAS and the SAHLPA was 0.82 (Table 3). Cronbach's alpha coefficients presented values in the optimal range: 0.85 for factor 1, 0.87 for factor 2 and 0.90 for the total scale. The average measures ICC calculated from the reliability post-mortem sub-study was 0.96 (CI 95% 0.92 to 0.99), revealing excellent inter-informant reliability.

**Table 3:** Spearman's correlation coefficients between the PCAS and construct criteria.

	PCAS	PCAS Subscale 1	PCAS Subscale 2	Educational Attainment	Occupation
**SAHLPA**	0.82^**^	0.79^**^	0.71^**^	0.66^**^	0.58^**^
**NEUROPSI Total Score**	0.73^**^	0.71^**^	0.53^**^	0.62^**^	0.55^**^
*Orientation*	0.36*	0.37*	0.35*	0.26*	0.22
*Attention*	0.64^**^	0.58^**^	0.50^**^	0.63^**^	0.51^**^
*Visuospatial Skills*	0.52^**^	0.46^**^	0.29*	0.50^**^	0.57^**^
*Memory*	0.47^**^	0.51^**^	0.29*	0.39*	0.31*
*Language*	0.74^**^	0.70^**^	0.64^**^	0.52^**^	0.53^**^
*Reading and Writing*	0.60^**^	0.62^**^	0.50^**^	0.48^**^	0.34*
*Conceptual Functions*	0.66^**^	0.65^**^	0.59^**^	0.49^**^	0.46^**^
*Motor Functions*	0.50^**^	0.46^**^	0.22	0.40*	0.49^**^

* p<0.05; **p<0.0001; Abbreviations: PCAS, Premorbid Cognitive Abilities Scale; SAHLPA, Short-Assessment of Health Literacy for Portuguese-Speaking Adults.

**Figure 3:**
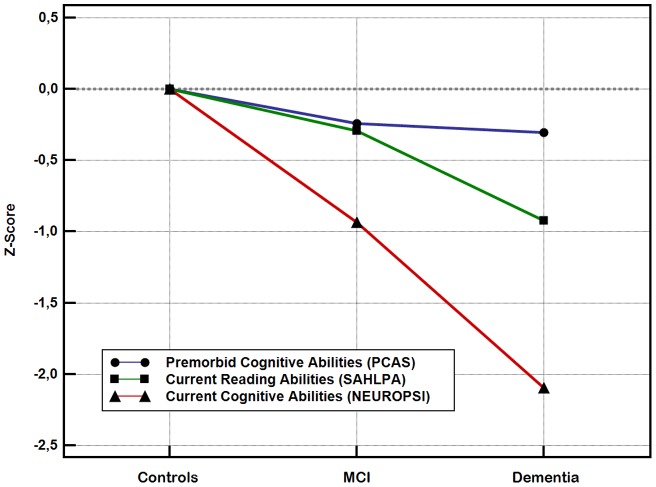
Between-group comparison of mean PCAS scores and construct criteria. Scores were transformed into z-scores to allow graphic comparison. Abbreviations: PCAS, Premorbid Cognitive Abilities Scale; SAHLPA, Short Assessment of Health Literacy for Portuguese-Speaking Adults; NEUROPSI, neuropsychological battery total score; MCI, Mild Cognitive Impairment.

As shown in Table 4, NEUROPSI total scores presented an approximately linear pattern of decline with increasing severity of cognitive impairment. Reading skills as measured by the SAHLPA were maintained in individuals with mild cognitive impairment, but were shown to decline in individuals with mild dementia. The PCAS scores were not significantly different across diagnostic categories (Figure 3).

**Table 4:** Between-group comparison of mean PCAS scores and construct variables.

	Controls (n = 72)	MCI (n = 33)	Dementia (n = 27)	P Value	Post-hoc test
**Premorbid Cognitive Abilities** (PCAS)	17.2 (6.8)	15.6 (7.0)	15.1 (8.0)	0.334*	
**Current Reading Abilities** (SAHLPA)	36.9 (10.2)	33.9 (13.0)	27.5 (15.0)	0.006^†^	C = M>D
**Current Cognitive Abilities** (NEUROPSI total score)	94.6 (16.0)	79.7 (17.8)	61.1 (20.2)	<0.001*	C>M>D

Data are shown as the mean (standard deviation). Abbreviations: MCI, Mild Cognitive Impairment; PCAS, Premorbid Cognitive Abilities Scale; SAHLPA, Short-Assessment of Health Literacy for Portuguese-Speaking Adults; * ANOVA; † Kruskal-Wallis test. “C>M” means that C is significantly higher than M.

## Discussion

To the best of our knowledge, the PCAS is the first instrument developed to estimate premorbid cognitive abilities in low-educated populations. By focusing on activities that are easily observable, with low economic demands and that are universal rather than culturally specific, the instrument is deemed to have good cross-cultural properties.

The overall scale and each of the subscales demonstrated adequate internal consistency, indicating a good level of correlation between items without redundancy. Previous validation studies have reported overall Cronbach's alpha values varying from 0.65 to 0.88 [16]–[18], [20]. The scale validated in this study presented a corresponding value of 0.90. This higher internal consistency can be attributed to the fact that PCAS is more focused on abilities that involve literacy and numeracy, while some existing instruments may have their internal consistency undermined by including remarkably diverse activities [17], [18].

Two studies have reported moderate to strong correlations between cognitive reserve questionnaires and reading tests, with coefficients of 0.45 and 0.62 [21], [19]. The correlation of the PCAS scores with reading skills as measured by the SAHLPA was remarkably high (r = 0.82). Again, this may have occurred because our instrument is focused on abilities that require reading and writing skills.

Previous studies described only weak to moderate correlations between cognitive reserve questionnaires and cognitive tests, with coefficients varying from 0.25 to 0.40 in participants with normal cognitive aging [17], [19]. Our study has found a correlation coefficient of 0.73 between PCAS and NEUROPSI total scores, with some of the domains presenting particularly high correlations, such as language (r = 0.74), conceptual functions (r = 0.66), and attention (r = 0.64). We hypothesize that the strength of association between such questionnaires and current cognitive performance may be higher in low-educated populations. The relationship between education and cognitive performance is not linear but, rather, is a negatively accelerated curve tending to a plateau [30]. It is plausible that a similar pattern would occur with questionnaires designed to estimate premorbid cognitive abilities.

In addition to the preceding hypothesis, four additional hypotheses can be invoked to explain the higher correlations with validity constructs that we found in our study. First, previous studies relied on self-reporting; instead we relied on information provided by a knowledgeable informant, which may be less affected by social desirability bias and recall bias associated with episodic memory impairment. Second, instead of rating the frequency of participation, we inquired about observable abilities with a yes/no endorsement. This simpler structure may have yielded more accurate answers. Additionally, in a cohort study with 379 older women, participation in a variety of activities was more predictive than frequency of participation for reductions in risk of incident impairment [38]. Third, although we tried to represent a variety of activities, we kept a focus on reading and writing skills. In a recent study conducted with older adults from Chicago, education and reading ability, but not leisure activities, provided the most accurate estimates of cognitive performance [39]. This finding supports the idea that the PCAS is focused on the most robust proxy measures of intelligence and cognitive reserve. Forth, informants were required to report the abilities of their relatives or friends 10 years ago. This may have attenuated recall errors because abilities are rated at a specific period in the patient's life. However, we conceive that adaptations of this criterion may yield good results. The method of rating the “best lifetime performance” is particularly appealing and could be tested in future studies.

The results also indicate that the PCAS is relatively resistant to the effects of cognitive decline, as suggested by the lack of a significant difference in scores across progressive levels of cognitive impairment. However, we cannot rule out a modest effect of cognitive impairment on the scale. The power of our sample was determined to be 0.72 for detecting a medium effect size (*f* = 0.25) but only 0.16 for detecting a small effect size (*f* = 0.10). A post hoc analysis with power set at 0.80 and alpha 0.05 showed us that a sample of 558 individuals would be necessary for the differences encountered between groups to reach statistical significance.

The sub-study conducted with 20 pairs of informants revealed excellent inter-informant reliability. The 95% CI lower limit for the ICC was above 0.90, indicating that although the sample was small, it had a sufficient size for the proposed aims. It is important to note that we evaluated reliability only between informants of normal or mildly impaired individuals. To what extent the value encountered will change in more severely impaired samples needs to be explored in future studies.

Some limitations of the study should be noted. First, the goal to develop a brief and easy-to-use scale may have introduced shortcomings, mainly by precluding the possibility of capturing more detailed information, such as the frequency of activities at each stage of life. Second, the scale did not include questions related to artistic pursuits, sporting, social life, and hobbies. It is possible that this feature may have significant drawbacks in populations of more developed regions, where leisure activities are widely incorporated into everyday life and might be a representative component of cognitive reserve. Third, development and validation procedures were conducted with data from the same sample. Further studies with different samples are needed to confirm the findings of this study. Fourth, our data indicating good inter-informant reliability compels us to suppose that the instrument would also present good test-retest reliability, but for practical reasons, it was not possible to evaluate this additional criterion in the present study.

We believe that there are two situations in which the PCAS can be particularly useful. First, it can be used as a premorbid intelligence estimate, to establish individually adjusted norms for neuropsychological tests. Second, it can be used as a cognitive reserve estimate, especially in research involving cognitively impaired subjects or in post-mortem research where subjects have had their brains donated, but did not have relevant data collected before death. Further studies will be important to confirm the properties and suitability of the PCAS in the contexts mentioned above.

In conclusion, the PCAS proved to be a simple and straightforward instrument that was readily understood. The findings of this study generally support good construct validity, internal consistency, inter-rater reliability, and stability across early stages of cognitive impairment.

## Supporting Information

Appendix S1 PCAS original version in Portuguese.
(DOCX)

Appendix S2 PCAS translated into English.
(DOCX)
