# Supplementary material for: Estimating Premorbid Cognitive Abilities in Low-Educated Populations
Source: PLoS One. 2013 Mar 21;8(3):e60084. doi: 10.1371/journal.pone.0060084 (PMC3605367; doi:10.1371/journal.pone.0060084)
Supplement: Appendix S1 — PCAS original version in Portuguese. (DOCX) [file pone.0060084.s001.docx]

| **ESCALA DE HABILIDADES COGNITIVAS PRÉ-MÓRBIDAS** | | |  | DATA: | | | | / / | |
| --- | --- | --- | --- | --- | --- | --- | --- | --- | --- |
|  | | | | |  |  | |  |  |
| NOME DO PACIENTE: |  | | | | | | | | |
|  |  | | | | | | | | |
| NOME DO INFORMANTE: |  | PARENTESCO: | | | | |  | | |
|  |  | | | |  |  |  | |  |
| ( ) preenchido pelo informante ( ) preenchido pelo examinador | | | | | | | | |  |

*► Tente lembrar-se de como o seu familiar ou amigo era 10 anos atrás*

*► Marque sim apenas se ele(a) conseguia fazer a tarefa sem qualquer ajuda*

**LEITURA**

**1.** Ele(a) conseguia ler e entender pequenas frases?

( ) SIM ( ) NÃO

**2.** Ele(a) conseguia ler e entender reportagens de revistas?

( ) SIM ( ) NÃO

**3.** Ele(a) conseguia ler e entender um livro inteiro?

( ) SIM ( ) NÃO

**4**. Ele(a) conseguia ler e entender uma receita médica?

( ) SIM ( ) NÃO

**ESCRITA**

**5.** Ele(a) conseguia fazer uma lista de compras?

( ) SIM ( ) NÃO

**6.** Ele(a) conseguia anotar um recado?

( ) SIM ( ) NÃO

**7.** Ele(a) conseguia preencher um formulário com dados pessoais?

( ) SIM ( ) NÃO

**8.** Ele(a) conseguia escrever uma carta para outra pessoa?

( ) SIM ( ) NÃO

**CÁLCULOS**

**9.** Ele(a) conseguia fazer contas simples de multiplicação (tabuada)?

( ) SIM ( ) NÃO

**10.** Ele(a) conseguia fazer contas de porcentagem (desconto em preços)?

( ) SIM ( ) NÃO

**Continua**

**USO DE RECURSOS TECNOLÓGICOS**

**11.** Ele(a) conseguia usar a calculadora para fazer contas simples?

( ) SIM ( ) NÃO

**12.** Ele(a) conseguia usar o caixa eletrônico para sacar dinheiro?

( ) SIM ( ) NÃO

**13**. Ele(a) conseguia usar o computador para digitar um texto e imprimir?

( ) SIM ( ) NÃO

**ENCONTRAR INFORMAÇÕES ESPECÍFICAS**

**14.** Ele(a) conseguia achar um número na lista telefônica e ligar?

( ) SIM ( ) NÃO

**15**. Ele(a) conseguia achar um novo local no mapa?

( ) SIM ( ) NÃO

**16**. Ele(a) conseguia achar informações sobre um aparelho no manual de instruções?

( ) SIM ( ) NÃO

**17**. Ele(a) conseguia achar informações sobre um remédio na bula?

( ) SIM ( ) NÃO

**HÁBITOS DE LEITURA**

**18**. Ele(a) costumava ler jornais ou revistas pelo menos uma vez por semana?

( ) SIM ( ) NÃO

**19.** Quantos livros ele(a) costumava ler por ano?

( ) Nenhum ( ) 3 a 4 livros por ano

( ) 1 a 2 livros por ano ( ) 5 livros por ano ou mais

**ESCOLARIDADE**

**20.** Quantos anos de estudo ele(a) completou?

( ) Nenhum ( ) 4 a 7 anos

( ) 1 ano ( ) 8 a 11 anos

( ) 2 a 3 anos ( ) 12 anos ou mais

**OCUPAÇÃO**

**21.** Que profissão ele(a) exerceu por mais tempo?

( ) Trabalho braçal não-qualificado em zona rural

( ) Trabalho braçal não-qualificado em zona urbana (tarefas simples e repetitivas)

( ) Trabalho braçal qualificado (tarefas específicas que exigem treinamento)

( ) Trabalho não-braçal de rotinas na área de serviço ou autônomo

( ) Trabalho intelectual, administrativo ou técnico com necessidade de formação superior
