# Supplementary material for: Estimating Premorbid Cognitive Abilities in Low-Educated Populations
Source: PLoS One. 2013 Mar 21;8(3):e60084. doi: 10.1371/journal.pone.0060084 (PMC3605367; doi:10.1371/journal.pone.0060084)
Supplement: Appendix S2 — PCAS translated into English. (DOCX) [file pone.0060084.s002.docx]

| **PREMORBID COGNITIVE ABILITIES SCALE (PCAS)** | | |  | TODAY’S DATE: | | | | / / | | | |
| --- | --- | --- | --- | --- | --- | --- | --- | --- | --- | --- | --- |
|  | | | | | |  |  | |  | |  |
| PATIENT’S NAME: |  | | | | | | | | | | |
|  | |  | | | | | | | | | |
| INFORMANT’S NAME: | |  | | | KINSHIP: |  | | | | | |
|  | |  | | | |  |  | | |  |  |
|  | | | | | | | | | | |  |

**► Try to remember what your relative or friend was like 10 years ago.**

**► Check yes only if he/she was able to perform the task without any help.**

**READING**

**1.** Was he/she able to read and understand short sentences?

( ) YES ( ) NO

**2.** Was he/she able to read and understand magazine reports?

( ) YES ( ) NO

**3.** Was he/she able to read and understand an entire book?

( ) YES ( ) NO

**4**. Was he/she able to read and understand a medical prescription?

( ) YES ( ) NO

**WRITING**

**5.** Was he/she able to write down a shopping list?

( ) YES ( ) NO

**6.** Was he/she able to take note of a message?

( ) YES ( ) NO

**7.** Was he/she able to fill out a form with personal data?

( ) YES ( ) NO

**8.** Was he/she able to write a letter for someone else?

( ) YES ( ) NO

**CALCULATION**

**9.** Was he/she able to solve simple problems involving multiplication (times table)?

( ) YES ( ) NO

**10.** Was he/she able to solve problems involving percentage (price discount)?

( ) YES ( ) NO

**Continued**

**USE OF TECHNOLOGY**

**11.** Was he/she able to use a calculator to solve simple problems?

( ) YES ( ) NO

**12.** Was he/she able to use an ATM machine to withdraw cash?

( ) YES ( ) NO

**13**. Was he/she able to use a computer to type and print text?

( ) YES ( ) NO

**FINDING SPECIFIC INFORMATION**

**14.** Was he/she able to find a number in the phone book and make a call?

( ) YES ( ) NO

**15**. Was he/she able to find a new location on a map?

( ) YES ( ) NO

**16**. Was he/she able to find information about a device in the instruction booklet?

( ) YES ( ) NO

**17**. Was he/she able to find information about a drug on package inserts?

( ) YES ( ) NO

**READING HABITS**

**18**. Did he/she use to read magazines or newspapers at least once per week?

( ) YES ( ) NO

**19**. How many books per year did he/she use to read?

( ) None ( ) 3 to 4 books per year

( ) 1 to 2 books per year ( ) 5 books per year or more

**EDUCATIONAL ATTAINMENT**

**20.** How many years of schooling did he/she complete?

( ) None ( ) 4 to 7 years

( ) 1 year ( ) 8 to 11 years

( ) 2 to 3 years ( ) 12 years or more

**OCCUPATION**

**21.** Which was his/her longest held occupation?

( ) Rural unskilled manual work

( ) Urban unskilled manual work (menial and repetitive tasks)

( ) Skilled manual work (specific tasks that require training)

( ) Routine non-manual employee or self-employed work

( ) Intellectual, administrator or higher-degree technician
